# Chaotic Analysis of the Electroretinographic Signal for Diagnosis

**DOI:** 10.1155/2014/503920

**Published:** 2014-06-15

**Authors:** Surya S. Nair, K. Paul Joseph

**Affiliations:** Electrical Engineering Department, National Institute of Technology, Calicut, Kerala 673601, India

## Abstract

Electroretinogram (ERG) is a time-varying potential which arises from different layers of retina. To be specific, all the physiological signals may contain some useful information which is not visible to our naked eye. However this subtle information is difficult to monitor directly. Therefore the ERG signal features which are extracted and analyzed using computers are highly useful for diagnosis. This work discusses the chaotic aspect of the ERG signal for the controls, congenital stationary night blindness (CSNB), and cone-rod dystrophy (CRD) classes. In this work, nonlinear parameters like Hurst exponent (HE), the largest Lyapunov exponent (LLE), Higuchi's fractal dimension (HFD), and approximate entropy (ApEn) are analyzed for the three different classes. It is found that the measures like HE dimension and ApEn are higher for controls as compared to the other two classes. But LLE shows no distinguishable variation for the three cases. We have also analyzed the recurrence plots and phase-space plots which shows a drastic variation among the three groups. The results obtained show that the ERG signal is highly complex for the control groups and less complex for the abnormal classes with *P* value less than 0.05.

## 1. Introduction

All physiological signals exhibit complex behavior which reflects the nonlinear dynamic properties of a biological system. Considering this, the use of nonlinear tools to exhibit the chaotic behavior may be a better approach to explore the nature of the electroretinographic signal. The randomness of the ERG signal does not allow any form of time-series prediction. The study of nonlinear dynamics can contribute to the understanding of the ERG signal and the underlying retinal processes [1, 2].

### 1.1. Electroretinogram (ERG)

Electroretinogram is the time-varying potential which arises from different retinal layers and is elicited by a brief flash of light stimuli. Contact lens type electrode which carries a silver chloride wire is used to record ERG clinically. The electrode is placed on the cornea and is in the shape of a cup filled with saline. The reference electrode is placed either on the earlobe, temple, or forehead. The amplitude of the ERG waveform is in the range of tenths of millivolt which depends upon the stimulating and physiological conditions [3].

**Figure 1:**
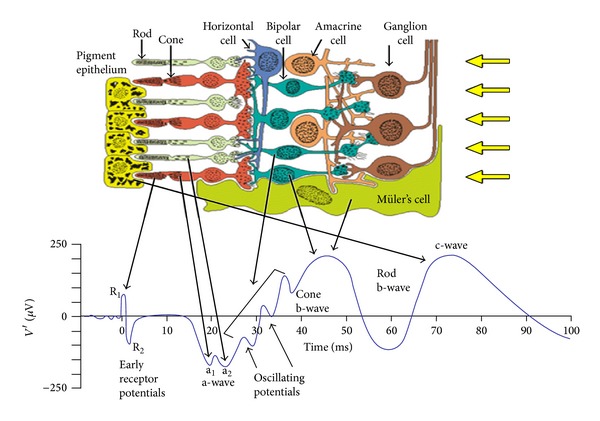
The cells of retina and the standard ERG waveform (courtesy: Jaakko Malmimo and Robert Plonsey, “Bioelectro-magnetism-Principles and Applications of Bioelectric and Biomagnetic fields”).

Figure 1 shows the cross-sectional view of human retina and the origin of signals from different layers of the retina. An ERG signal comprises of early receptor potential (ERP) *R*_1_ and *R*_2_, *a*-wave (*a*_1_ and *a*_2_), oscillatory potentials, *b*-wave (cone *b*-wave and rod *b*-wave), and *c*-wave. The initial changes in the photo pigment molecules of the photoreceptor cells (cone cells and rod cells) due to the brief flash of light stimulus will give rise to a positive *R*_1_ deflection followed by *R*_2_ deflection which forms the ERP. It is then followed by a late receptor potential (LRP) after a 2 ms delay which forms the main portion of the *a*-wave and is a corneo-negative wave. The *a*-wave comprises of two negative dips *a*_1_ and *a*_2_ which shows the contribution of cone cells and rod cells, respectively. It lasts for about 30 ms. These cone cells and rod cells can be separated by applying appropriate stimuli. A dim-blue light with the dark background extracts a rod ERG and a bright red light with the light adapted background will give a cone ERG. The Muller cells in the inner retina which contribute to the *b*-wave are a corneo-positive wave. Muller responses can be obtained either from the cone cells or from the rod cells separately. The oscillatory potentials which occur in the rising edge of the *b*-wave are small amplitude wavelets that reflect the activity of amacrine cells of the inner retinal layers. The *c*-wave which is generated by the retinal pigment epithelium (RPE) as a result of interaction with the rod cells is a slower positive wave [3].

### 1.2.  Recording of the ERG Signal

International Standards for Clinical Electrophysiology of Vision (ISCEV) sets the recording of the ERG signal in five different steps.

#### 1.2.1. Rod Response

The patient is dark adapted for at least 20 minutes. The standard stimulus of dim-white flash (2 seconds between the flash) of 2.5 log units or blue stimulus is given. It is the first signal measured during the ERG recording [4].

#### 1.2.2. Maximal Response

The combined response of both cone cells and rod cells gives maximal response and is produced by the white standard flash (10 seconds between the flashes) [4].

#### 1.2.3. Oscillatory Potentials

Oscillatory potentials or OP are obtained either from light adapted eye (1.5 seconds between flashes) or from dark adapted eye (15 seconds between flashes) using white standard flash. The frequency of interest is set by the band pass filter and the lowest frequency range is 75–100 Hz and 300 Hz and above at the higher end [4].

#### 1.2.4. Cone Response

Before recording the cone response, the patient should be light adapted for at least 10 minutes. The flash used is white or bright red in colour and 0.5 seconds between the flashes is the interval given [4].

#### 1.2.5. 30 Hz Flicker Response

Here the flicker type stimulus is used and flashes are given at the rate of 30 stimuli per second. Rods follow the flickering light up to 12–17 Hz and cones will follow up to 60–70 Hz [4].

### 1.3. Literature Study

The first remarkable work done in the field of ERG signal analysis by Bornschein et al. was the study of electroretinography in normal, colour-blind, and night-blind subjects under various states of adaptation with varying stimuli [5]. A series of work followed by Barraco et al. shows the three-frequency range of occurrence between 20 and 200 Hz [1] and also analyzed the time-frequency characteristics of the *a*-wave in congenital stationary night blindness (CSNB) patients [2, 6]. Study on the basis of principal component analysis and wavelet analysis was used to visualise the time domain features and wavelet features (Rogala and Brykalski) [7]. Another work reported in the area of ophthalmology is multifocal ERG analysis using wavelet transform by Miguel et al. for the diagnosis of glaucoma [8, 9]. Nair and Paul Joseph have analysed the ERG signal using wavelets and entropy analysis [10]. Study of Crevier and Meister showed that period-doubling occurs in the nonlinear dynamical system and observation of chaotic behavior in the nervous system is the period-doubling route to chaos in flicker vision of the ERG [11]. Molaie et al. showed that the parameters like flash frequency and contrast have a greater impact on the recorded ERG signals which cause bifurcations resulting in a period-doubling and the work defines neural network to be a powerful tool for modeling highly chaotic behavior in the nervous system [12].

To the best of our knowledge, no work is done on the nonlinear aspect of the ERG signal. Here, in this work, we are analysing the ERG signal of controls, congenital stationary night blindness (CSNB), and cone-rod dystrophy (CRD) with the nonlinear chaotic perspective of analysis.

### 1.4. Organisation of Work

The organisation of work includes the following subsections. In “Data Acquisition” Section we present the details of the acquisition of ERG recordings and various pathological diseases affecting the eye. “Analysis Methods” provides a brief overview of various characteristic measures like Hurst exponent, Lyapunov exponent, approximate entropy, fractal dimension, and recurrence plots for various cases. Following that the results are included. Final conclusion and discussion of the study are also reported.

## 2. Data Acquisition

Acquisition of the recordings of the ERG signal is performed using TOMEY EP 1000 version 3.0.4 from 15 control subjects, 20 subjects with congenital stationary night blindness (CSNB) type I, 15 subjects with CSNB type II, 35 subjects with cone-rod dystrophy also called as retinitis pigmentosa, among which 15 subjects are of typical RP (retinitis pigmentosa), 10 subjects of early onset RP, and 10 subjects of late stage RP. From the five steps of the recording of ERG signals, we are analyzing only the maximum response and 30 Hz flicker response from the above subjects. Patient data were collected from Little Flower Hospital and Research Centre, India, with the proper consent from the clinicians. Brief descriptions of the pathologies analyzed are given below.

### 2.1. Controls

The normal amplitude range of rod response is 140–250 *μ*V with the implicit time of 80–90 ms, maximum response ranges from 250 to 500 *μ*V amplitude, and implicit time is 45 ms. Cone response amplitude is 100–180 *μ*V with the implicit time of 32 ms and flicker response amplitude is 50 *μ*V approximately.

### 2.2. Congenital Stationary Night Blindness (CSNB)

It is an X-linked retinal disorder with abnormal nocturnal vision. It has two forms depending upon the severity: complete form CSNB type I and an incomplete form CSNB type II. The main difference between the two is in the complete form; there are no measurable rod cells, whereas in the incomplete form some response is obtained due to the rod cells. In the complete form, cone activity is also affected [13, 14].

### 2.3. Cone-Rod Dystrophy (CRD) or Retinitis Pigmentosa (RP)

CRD also called as retinitis pigmentosa is an inherited retinal dystrophy with retinal pigment deposits visible on fundus examinations. The *a*-wave and *b*-wave amplitude are reduced. It is again classified into three types, namely, typical RP, early onset RP, and late stage RP. In the first type, the symptom is night blindness. In the second type, macular involvement occurs early and there is an involvement of rod cells which supports the diagnosis. In the third type, there is a decrease in the visual acuity and also macular involvement. The appearance of night blindness or loss of central vision supports the diagnosis [15, 16].

## 3. Analysis Methods

In this work various characteristic measures like Hurst exponent, Largest Lyapunov exponent, Higuchi's fractal dimension, approximate entropy, and recurrence and phase-space plots are analyzed. A brief description of each of the parameters is given below.

### 3.1. Hurst Exponent

Hurst exponent is used to evaluate the long range dependence of data and its degree in a time series. Hurst exponent is the measure of smoothness of a fractal time series. It can also be defined as

(1) $$\begin{array}{c} H=\frac{\log\left(R/S\right)}{\log\left(T\right)}, \end{array}$$

where *T* is the duration of the ERG sample of data and *R*/*S* is the value of rescaled range. If *H* = 0.5, the time series acts as a random walk. If *H* < 0.5, the time series covers less distance than a random walk. If *H* > 0.5, the time series covers larger distance than a random walk.

To estimate the Hurst exponent, the dependence of the rescaled range on the time span *n* is first estimated. A time series of length *N* is divided into shorter time series of length *n* = *N*, *N*/2, *N*/4…. The average rescaled range is computed for each “*n*” [17]. Step-by-step explanation of rescaled range calculation is as follows.

(i) Calculate the mean:
  (2) $$\begin{array}{c} m=\frac{1}{n}\overset{n}{\underset{i=1}{\sum }}X_{i},\;\text{where}\;\;X=X_{1},X_{2},\ldots X_{n}. \end{array}$$
(ii) Create a mean-adjusted time series:
  (3) $$\begin{array}{c} Y_{t}=X_{t}-m\;\text{for}\;\;t=1,2,\ldots n. \end{array}$$
(iii) Compute the cumulative deviate series say *Z*:
  (4) $$\begin{array}{c} Z_{t}=\overset{t}{\underset{i=1}{\sum }}Y_{i}\;\text{for}\;\;t=1,2,\ldots n. \end{array}$$
(iv) Compute the range *R*:
  (5) $$\begin{array}{c} R\left(n\right)=\max\left(Z_{1},Z_{2,\ldots }Z_{n}\right)-\min\left(Z_{1},Z_{2,\ldots }Z_{n}\right). \end{array}$$
(v) Compute *S*, standard deviation,
  (6) $$\begin{array}{c} S\left(n\right)=\sqrt{\frac{1}{n}}\overset{n}{\underset{i=1}{\sum }}{\left(X_{i}-n\right)}^{2}. \end{array}$$
(vi) Calculate the rescaled range *R*(*n*)/*S*(*n*) and average over all time series of “*n*.”

### 3.2. The Largest Lyapunov Exponent (LLE)

Lyapunov exponent is used to distinguish between the periodic and chaotic signals. In phase space, the trajectories of chaotic dynamics follow typical patterns. It is the rate at which the neighboring trajectories separate from each other. A zero exponent indicates that the orbits maintain their relative positions. A negative exponent shows that the orbits approach a common point and the positive exponent shows that they are on chaotic attractor.

For any two points in a space say *X*_0_ and *X*_0_ + Δ*x*_0_, each of the points generates their own orbit in the space using a set of equations, where Δ*x* is the separation between the two orbits. This separation Δ*x* is the function of initial value Δ*x*(*X*_0_, *t*). Then the Lyapunov exponent *λ* is given by

(7) $$\begin{array}{c} \lambda =\underset{t\to \infty }{\lim}\frac{1}{t}\ln\frac{\left|\Delta x\left(X_{0},t\right)\right|}{\left|\Delta X_{0}\right|}. \end{array}$$

The largest Lyapunov exponent is computed by the least square fit to average line and is defined as

(8) $$\begin{array}{c} y\left(n\right)=\frac{1}{\Delta t}\langle \ln\left(d_{i}\left(n\right)\right)\rangle , \end{array}$$

where *d*_*i*_(*n*) is distance between *i*th phase-space point and its nearest neighbors at *n*th time and 〈·〉 is the average overall phase-space points [18–20].

### 3.3. Approximate Entropy (ApEn)

Approximate entropy which is applied to the relatively short and noisy data is the logarithmic likelihood that the sample points which are close to each other will be same for the next comparison with a longer pattern. Smaller ApEn value shows that the signal is deterministic and higher ApEn value shows that the signal is random [21, 22]:

(9) ApEn(m,r,N) =1N−m∑i=1N−mlog⁡(Cim+1(r))−(1N−m+1)  ×∑i=1N−m+1log⁡⁡(Cim(r)),

where *C*_*i*_^*m*^(*r*) is correlation integral, *m* is pattern length, and *r* is effective filter [23, 24].

### 3.4. Fractal Dimension (FD)

In traditional geometry, the Euclidean dimension of an object is referred to as the number of directions each differential of the object occupies in a space. The FD is used to provide a measure of how much space is occupied by an object between the Euclidean dimensions. In this work, we are using Higuchi's algorithm for the analysis [25].

Let *x*(1), *x*(2) … *x*(*N*), be the time series to be analyzed. Let *x*_*m*_^*k*^ be the *k* new time series:

(10) xmk={x(m),x(m+k),x(m+2k),   …x(m+[N−mk]k)},

where *m* = 1,2 … *k* and *m* is initial time value and *k* is the discrete time interval.

For each *x*_*m*_^*k*^, *L*_*m*_(*k*) (length) is computed by

(11) $$\begin{array}{c} L_{m}\left(k\right)=\overset{\left[a\right]}{\underset{i=1}{\sum }}\left|x\left(m+ik\right)-x\left(m+\left(i-1\right)k\right)\right|\frac{\left(N-1\right)}{\left[a\right]k}, \end{array}$$

where *N* is the total length of data *x*, (*N* − 1)/[*a*]*k* is normalization factor, and *a* is (*N* − *m*)/*k*.

This procedure is repeated for different values of *k* ranging from 1 to *k*_max⁡_, obtaining the average length. Fractal dimension is the slope of the least square linear best fit of the graph ln⁡(*L*_*m*_(*k*)) versus ln⁡(1/*k*).

### 3.5. Recurrence Plots (RP)

Recurrence plot is a visualization technique which is used to detect hidden dynamical patterns and correlations in the data. In general, the RP reveals all those times at which the phase-space trajectory visits roughly the same area in the phase space. Suppose we have the time series {*X*_*i*_}_*i*=1_^*N*^ representing the trajectory in the phase space with *X*_*i*_ ∈ *R* ^*d*^. RP is based on the following equation:

*R*_*i*,*j*_ = Θ(∑−||*X*_*i*_ − *X*_*j*_||), *i*, *j* = 1,2,…*N*.  Θ = Heaviside function. ||·|| = norm. ∑ = predefined threshold.

Recurrence plots are mainly of four types, homogenous, drift, periodic, and disrupted. Homogenous RP are of stationary type and relaxation time is short with respect to the time spanned by the RP. Oscillating systems have RP (periodic) with diagonal lines and checkerboard structures. Slowly varying parameters with brightened RP at upper left and lower right corners are drift RP. Extreme events or sudden changes in the dynamics produce disrupted recurrence plots [26].

### 3.6. Surrogate Data Analysis

Surrogate data analysis is used to check the nonlinearity in the original data. Surrogate data is generated by phase randomizing the original dataset. The surrogate data has the same mean, variance, autocorrelation function, and similar spectral properties as of original data but phase relations are different. 15 surrogate series were generated from each original data series. Statistical significance is measured by comparing the experimental data with the surrogate data. If both results differ more than 50%, then the null hypothesis is rejected and it shows that the original data is nonlinear [27].

## 4. Results

Nonlinear parameters like Hurst exponent, approximate entropy, Higuchi's fractal dimension, and the largest Lyapunov exponent are calculated for 30 Hz flicker signal and oscillatory potential of ERG signal. Tables 1 and 2 show the unique parameters in different cases along with the *P* values.

The Hurst exponent which is in the range of 0-1 is used to measure the long range dependence of a time series. In each of the cases shown in Tables 1 and 2, the HE varies with a *P* value less than 0.05. Approximate entropy which measures the disorder or predictability of the ERG signal shows higher values for control and the range of values reduces with each pathological case. The time lag used is 1 with *m* value of 2. For ApEn *r* value is taken as 15% of the standard deviation. The *r* value is selected on the basis of previous studies which indicate good statistical validity [22]. HFD also showed reduced values for abnormal cases due to reduced rhythmic variation. Dimension value is taken as 9 with the delay of 1 in our study.

**Figure 2:**
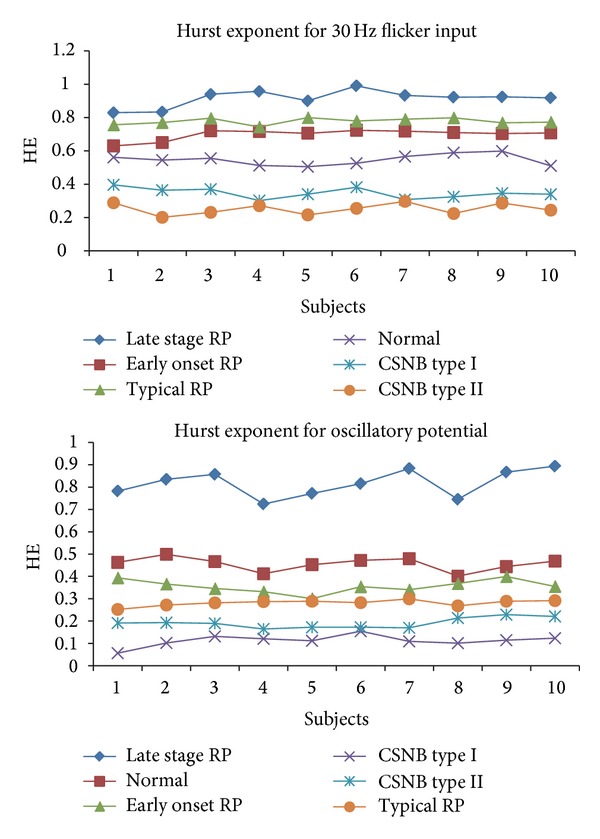
Variation of HE for six cases.

**Figure 3:**
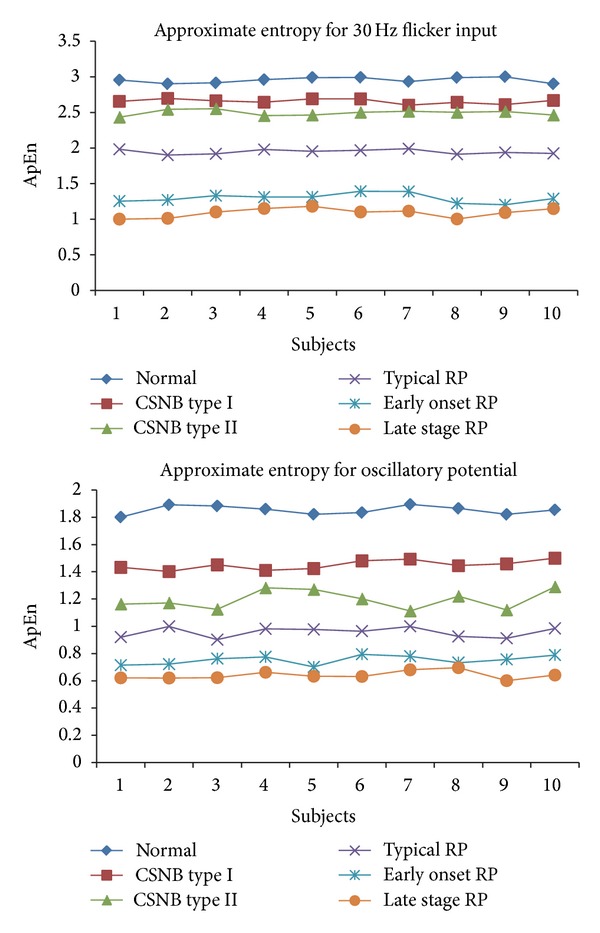
Variation of ApEn for six cases.

**Figure 4:**
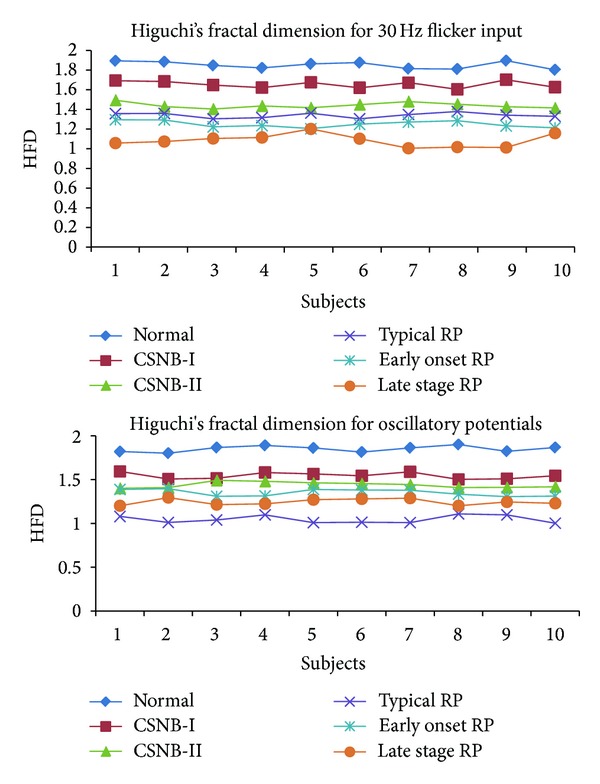
Variation of HFD for six cases.

Figures 2, 3, and 4 show the Hurst exponent, LLE, and ApEn for the ERG signal. In our analysis, LLE value is a positive exponent which means that the signal is chaotic but the value is not distinguishable for different cases with the *P* value greater than 0.05.

**Figure 5:**
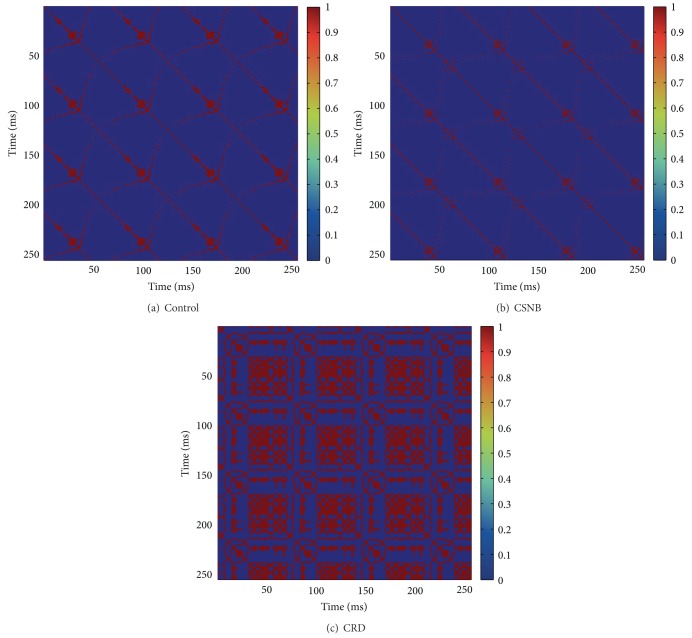
Recurrence plots for 30 Hz flicker input.

**Figure 6:**
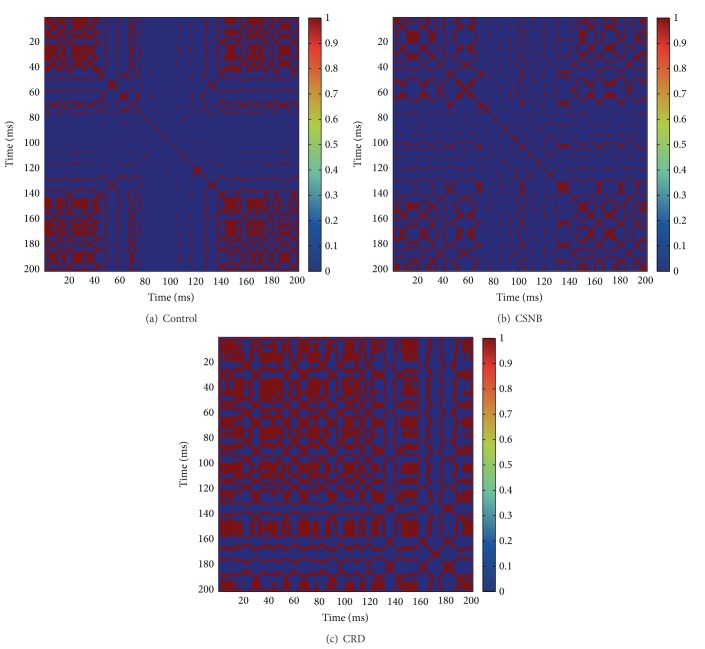
Recurrence plots for oscillatory potential input.

Recurrence plot shows unique pattern in the case of control, CSNB, and CRD groups. In cone-rod dystrophy, also known as retinitis pigmentosa, recurrence plots show more squares in the case of both 30 Hz flicker input and oscillatory potential input (Figures 5 and 6). It indicates that, in CRD groups, a rhythmic variation causes periodicity and more patches of colors indicate variation of the signal.

## 5. Discussion and Conclusion

In this paper we have demonstrated and introduced the nonlinear analysis as a tool to evaluate the electroretinographic signal. All biological signals are random in nature and this randomness does not provide any time domain analysis and prediction. A nonlinear deterministic chaos theory and chaotic indicators are used for the analysis of ERG signal. In our work, quantitative schemes such as Hurst exponent, Lyapunov exponent, Higuchi's fractal dimension, and approximate entropy are analyzed. Qualitative analysis like recurrence plots and phase-space plots are also computed for controls and different pathological groups. From our work, it can be concluded that all the nonlinear parameter values are higher for the control subjects and smaller values in the case of CSNB and CRD group which clearly shows the reduction of rhythmic variation in the pathological cases. In all the six cases (normal, CSNB I, CSNB II, typical RP, early onset RP, and late stage RP) the parameters like Hurst exponent, ApEn, HFD, and average recurrence show distinguishable numerical information from the ERG signal. From our study the largest Lyapunov exponent (LLE) shows positive value in all cases indicating the confirmation of the chaotic nature of the ERG signal. But LLE is not distinguishable for different groups. Recurrence plots and phase-space plots provide the visual inspection tool to assess the time evolution and the frequency of their recurrences. In our previous work, we have analyzed the ERG signal using wavelet analysis [10]. The method described in this paper is the analysis of the same signal from the nonlinear perspective of view. No work has yet been reported till now on the nonlinear aspect of the ERG signal analysis. Current work provides an excellent method for more advanced studies in the field of ophthalmology. Analysis and methods are individual dependent and the existing literatures prove that nonlinear methods are more effective in the analysis of biomedical signals like EEG (electroencephalogram) and HRV (heart rate variability) analysis.

## Figures and Tables

**Table 1:** Nonlinear parameter values in different cases for 30 Hz flicker ERG signal.

Parameters	Controls	CSNB I	CSNB II	Typical RP	Early onset RP	Late stage RP	*P* value
HE	0.5468 ± 0.042	0.3467 ± 0.04	0.2513 ± 0.0214	0.7768 ± 0.012	0.6987 ± 0.0314	0.9144 ± 0.0654	0.0051
ApEn	2.9527 ± 0.0301	2.6553 ± 0.0425	2.4934 ± 0.028	1.9464 ± 0.01	1.2970 ± 0.006	1.0904 ± 0.043	0.0124
HFD	1.8483 ± 0.0401	1.6526 ± 0.0912	1.4366 ± 0.0048	1.3370 ± 0.03	1.2464 ± 0.0062	1.0809 ± 0.012	0.123
LLE	0.4823 ± 0.0031	0.457 ± 0.001	0.41 ± 0.004	0.43 ± 0.0015	0.401 ± 0.007	0.422 ± 0.0017	0.067

**Table 2:** Nonlinear parameter values in different cases for oscillatory potential of ERG signal.

Parameters	Controls	CSNB I	CSNB II	Typical RP	Early onset RP	Late stage RP	*P* value
HE	0.4551 ± 0.033	0.1120 ± 0.0112	0.1909 ± 0.0241	0.2805 ± 0.0306	0.3548 ± 0.016	0.8168 ± 0.0212	0.002
ApEn	1.8526 ± 0.005	1.4491 ± 0.0041	1.1947 ± 0.0203	0.95608 ± 0.052	0.7522 ± 0.004	0.6405 ± 0.031	0.034
HFD	1.8501 ± 0.0301	1.5448 ± 0.0165	1.4383 ± 0.026	1.0098 ± 0.003	1.3142 ± 0.0046	1.2696 ± 0.011	0.106
LLE	0.7090 ± 0.0015	0.7577 ± 0.0009	0.7134 ± 0.002	0.7043 ± 0.0052	0.741 ± 0.0064	0.732 ± 0.0041	0.079

## References

[1] Barraco R, Bellomonte L, Brai M, Perrano Adorno D (2008). Functional analysis of Normal and CSNB a-wave ERG component. *IFMBE Proceedings*.

[2] Barraco R, Bellamonte L, Brai M (2007). Time-frequency behaviour of the a-wave of the human electroretinogram. *IFMBE Proceedings*.

[3] Malmimo J, Plonsey R (1995). *Bioelectromagnetism: Principles and Applications of Bioelectric and Biomagnetic Fields*.

[4] Marmor MF, Holder GE, Seeliger MW, Yamamoto S (2004). Standard for clinical electroretinography (2004 update). *Documenta Ophthalmologica*.

[5] Bornschein H, Goodman G, Gunkel RD (1957). Temporal aspects of the human electroretinogram; a study of the implicit time-amplitude relationship of the b-wave. *A.M.A. Archives of Ophthalmology*.

[6] Barraco R, Persano Adorno D, Brai M (2011). ERG signal analysis using wavelet transform. *Theory in Biosciences*.

[7] Rogala T, Brykalski A (2005). Wavelet feature space in computer-aided electroretinogram evaluation. *Pattern Analysis and Applications*.

[8] Miguel JM, Boquete L, Ortega S, Cordero CA, Barea R, Blanco R (2012). MfERG_LAB: software for processing multifocal electroretinography signals. *Computer Methods and Programs in Biomedicine*.

[9] Miguel-Jiménez JM, Ortega S, Boquete L, Rodríguez-Ascariz JM, Blanco R (2011). Multifocal ERG wavelet packet decomposition applied to glaucoma diagnosis. *BioMedical Engineering Online*.

[10] Nair SS, Paul Joseph K (2014). Wavelet based electroretinographic signal analysis for diagnosis. *Biomedical Signal Processing and Control*.

[11] Crevier DW, Meister M (1998). Synchronous period-doubling in flicker vision of salamander and man. *Journal of Neurophysiology*.

[12] Molaie M, Falahian R, Gharibzadeh S, Jafari S, Sprott JC (2014). Artificial neural networks: powerful tools for modeling chaotic behavior in the nervous system. *Frontiers in Computational Neuroscience*.

[13] Zeitz C, Forster U, Neidhardt J (2007). Night blindness-associated mutations in the ligand-binding, cysteine-rich, and intracellular domains of the metabotropic glutamate receptor 6 abolish protein trafficking. *Human Mutation*.

[14] Zeitz C (2007). Molecular genetics and protein function involved in nocturnal vision. *Expert Review of Ophthalmology*.

[15] Hamel CP (2007). Cone rod dystrophies. *Orphanet Journal of Rare Diseases*.

[16] Moore AT (1992). Cone and cone-rod dystrophies. *Journal of Medical Genetics*.

[17] Dangel S, Meier PF, Moser HR, Plibersek S, Shen Y (1999). Time series analysis of sleep EEG. *Computer Assisted Physics*.

[18] Wolf A, Swift JB, Swinney HL, Vastano JA (1985). Determining Lyapunov exponents from a time series. *Physica D: Nonlinear Phenomena*.

[19] Das A, Das P, Roy AB (2002). Applicability of Lyapunov exponent in EEG data analysis. *Complexity International*.

[20] Rosenstein MT, Collins JJ, De Luca CJ (1993). A practical method for calculating largest Lyapunov exponents from small data sets. *Physica D: Nonlinear Phenomena*.

[21] Acharya UR, Joseph KP, Kannathal N, Lim CM, Suri JS (2006). Heart rate variability: a review. *Medical and Biological Engineering and Computing*.

[22] Pincus SM (1991). Approximate entropy as a measure of system complexity. *Proceedings of the National Academy of Sciences of the United States of America*.

[23] Richman JS, Moorman JR (2000). Physiological time-series analysis using approximate and sample entropy. *American Journal of Physiology: Heart and Circulatory Physiology*.

[24] Pincus SM, Goldberger AL (1994). Physiological time-series analysis: what does regularity quantify?. *American Journal of Physiology: Heart and Circulatory Physiology*.

[25] Klonowski W, Olejarczyk E, Stepien R Sleep-EEG analysis using Higuchi’s fractal dimension.

[26] Mohebbi M, Ghassemian H (2011). Structures of the recurrence plot of heart rate variability signal as a tool for predicting the onset of paroxysmal atrial fibrillation. *Journal of Medical Signals and Sensors*.

[27] Theiler J, Eubank S, Longtin A, Galdrikian B, Doyne Farmer J (1992). Testing for nonlinearity in time series: the method of surrogate data. *Physica D: Nonlinear Phenomena*.

